# Pollen and spores as biological recorders of past ultraviolet irradiance

**DOI:** 10.1038/srep39269

**Published:** 2016-12-15

**Authors:** Phillip E. Jardine, Wesley T. Fraser, Barry H. Lomax, Mark A. Sephton, Timothy M. Shanahan, Charlotte S. Miller, William D. Gosling

**Affiliations:** 1 School of Environment, Earth and Ecosystem Sciences, The Open University, Walton Hall, Milton Keynes, MK7 6AA, UK; 2 Geography, Department of Social Sciences, Oxford Brookes University, Oxford OX3 0BP, UK; 3 Agriculture and Environmental Science, University of Nottingham, Sutton Bonington Campus, Leicestershire, LE12 5RD, UK; 4 Department of Earth Sciences & Engineering, Imperial College London, South Kensington, London SW7 2AZ, UK; 5 Department of Geological Sciences, The University of Texas at Austin, 1 University Station C9000, Austin, Texas 78712, USA; 6 MARUM,University of Bremen, Leobener Str., D-28359 Bremen, Germany; 7 Palaeoecology & Landscape Ecology, Institute of Biodiversity & Ecosystem Dynamics (IBED), University of Amsterdam, 1090 GE Amsterdam, The Netherlands

## Abstract

Solar ultraviolet (UV) irradiance is a key driver of climatic and biotic change. Ultraviolet irradiance modulates stratospheric warming and ozone production, and influences the biosphere from ecosystem-level processes through to the largest scale patterns of diversification and extinction. Yet our understanding of ultraviolet irradiance is limited because no method has been validated to reconstruct its flux over timescales relevant to climatic or biotic processes. Here, we show that a recently developed proxy for ultraviolet irradiance based on spore and pollen chemistry can be used over long (10^5^ years) timescales. Firstly we demonstrate that spatial variations in spore and pollen chemistry correlate with known latitudinal solar irradiance gradients. Using this relationship we provide a reconstruction of past changes in solar irradiance based on the pollen record from Lake Bosumtwi in Ghana. As anticipated, variations in the chemistry of grass pollen from the Lake Bosumtwi record show a link to multiple orbital precessional cycles (19–21 thousand years). By providing a unique, local proxy for broad spectrum solar irradiance, the chemical analysis of spores and pollen offers unprecedented opportunities to decouple solar variability, climate and vegetation change through geologic time and a new proxy with which to probe the Earth system.

Earth’s energy budget is ultimately governed by incoming solar irradiance^1^. Ultraviolet (UV) radiation comprises just 8% of total solar irradiance (TSI)^2^, but exerts a primary control on atmospheric dynamics via stratospheric warming and modulation of ozone abundance^2^,^3^,^4^. UV (and in particular UV-B) is a key stress factor on the biosphere and impacts upon ecosystem-level processes such as competition and nutrient cycling^5^,^6^. UV-B radiation induces genome instability by directly damaging DNA^6^,^7^, and has been invoked as a cause of both diversification and extinction during the Phanerozoic (the last 540 Ma)^8^,^9^.

Empirical knowledge of the role of UV in driving past climatic and biological change is absent owing to the lack of a palaeo-UV proxy that can be demonstrably applied over geologic timescales. Indicators of solar activity such as counts of sunspot numbers and cosmogenic radionuclides (^10^Be and ^14^C) are limited to timescales of 10^2^ to 10^3^ years, respectively^1^, but the relationship between solar activity and UV flux is uncertain^4^. Calculated TSI variability across Milankovitch orbital cycles^10^ provides an expected pattern for incoming UV radiation over timescales of 10^4^ to 10^7^ years, but cannot detect short-term, surface-level departures from the orbital signal, for example those due to ozone thinning^9^, volcanic events^11^, dust clouds from bolide impacts^12^, or continental uplift^13^. A direct proxy for palaeo-UV flux would therefore provide both an indicator of UV as a climatic, environmental, and evolutionary forcing mechanism, and a recorder of atmospheric change across some of the major perturbations in Earth history.

Sporomorphs (pollen and spores), the reproductive vectors of land plants, have a highly recalcitrant biogeochemistry, enabling their preservation and fossilisation. The outer wall (exine) is primarily made of sporopollenin, a biopolymer that is highly resistant over geological timescales^14^. Within sporopollenin, protection from the deleterious effects of UV-B is provided by the phenolic compounds *para*-coumaric acid and ferulic acid^15^,^16^. These UV absorbing compounds (UACs) covary with UV irradiance in a range of modern settings^13^,^15^,^16^,^17^,^18^,^19^, demonstrating that plants actively control the chemical composition of sporopollenin with up-regulation in UACs in response to elevated UV doses^16^. The high preservation potential of sporopollenin, and the resultant high abundances of sporomorphs in the fossil record, suggests that a UAC-based proxy could be used to reconstruct UV flux in deep time settings^16^,^20^. Critically, experimental studies have shown that sporopollenin chemistry is resistant to moderate levels of oxidation^21^ and thermal maturation^22^, and its stability has been demonstrated in samples over 400 million years old^23^. While there is a suggested relationship between sporopollenin UAC concentrations and modelled solar influx through the last 9.5 kyr^19^, the stability and recovery of this proxy has yet to be thoroughly tested over longer periods of time. There is considerable uncertainty with regard to how UV flux varies through time^4^, but over longer timescales orbitally-modulated variations in TSI should be the major control on UV flux^24^, and UAC concentrations should track modelled TSI. A well-constrained modern test environment for the UAC-based proxy is provided by variation in Earth’s latitude. Success in discriminating UAC responses to latitude would allow confident extension of the proxy to track orbitally-driven TSI through geological time.

Today both UV and TSI increase with decreasing latitude^6^. If our proxy is to be used effectively to track major perturbations in UV flux in the geologic record, then we must be able to demonstrate an unequivocal response in UAC contents with latitude. Using FTIR microspectroscopy we have measured UACs in *Lycopodium* (club moss) spores from 217 individual plants spanning a TSI range of 200 W/m^2^ (see Methods). *Lycopodium* is an ideal taxon for assessing latitudinal trends in UAC concentrations because (a) it is geographically and latitudinally widespread; (b) its chemistry is well-understood from previous research on UV perception and reconstruction^13^,^15^,^17^,^18^,^21^; and (c) the morphology of the plants, with sporangia facing upwards, means that the developing spores receive a clear UV signal. Encouragingly there is a strong positive relationship between UAC abundance in *Lycopodium* spores and TSI flux (Fig. 1 and Supplementary Tables S1, S2), demonstrating that TSI variation can be recovered from sporopollenin chemistry. Furthermore, our data are consistent with a recently published latitudinal UAC gradient based on *Pinus* pollen (Fig. 1)^19^, confirming the cross-taxa applicability of a novel biochemical proxy that tracks changes in TSI radiation. Our data are also supported by previous work showing that the mechanism behind the perception and subsequent upstream regulation of plant responses to UV-B is controlled at the genetic level across widely spaced phylogenetic groups^25^,^26^. Taken together these multiple lines of evidence suggest that the underlying UV-B response mechanisms and their expression are highly conserved, indicating that a TSI signal can be recovered from a large variety of taxa.

To test the validity of the UV proxy through time, we need long, independently dated sporomorph records that can be linked to orbital TSI patterns. One such record is Lake Bosumtwi in Ghana (06°32′N, 01°25′W; Supplementary Fig. S1)^27^. Today Lake Bosumtwi is surrounded by moist semi-deciduous tropical forest^28^,^29^, but for most of the last 500 kyr it was dominated by grasslands^28^. The evolutionary conservative nature of the plant UV-response means that we can target site-specific appropriate (i.e. abundant) taxa, such as grasses. Interpretation of the TSI signal requires an understanding of seasonal pollen production (see Methods). Maximum flowering season^30^ TSI occurs in September (Supplementary Fig. S1), and we therefore use modelled September TSI^32^ (Fig. 2a) as an expectation to test the grass pollen UAC concentrations against.

Sporopollenin UAC content varies across the two sampled grassland intervals at Lake Bosumtwi (Fig. 2b and Supplementary Tables S3, S4). Both solar inputs and shading from higher stature vegetation^17^ will control UAC concentrations; a multiple regression model with both modelled TSI and Poaceae proportion as a proxy for habitat openness gives a significant positive relationship (Fig. 3a) (see Methods). Generalized Additive Model (GAM) smoothers show pronounced cyclicity with a wavelength of approximately 20 kyr, which is in line with the precessional component of the Milankovitch cycle; 19–21 kyr^31^. Regression of the GAM-predicted UAC values gives further statistical support to our findings (Fig. 3b). In addition, the association between UACs and TSI holds when age-depth model uncertainty is taken into account (Fig. 4 and Supplementary Fig. S2). Although these pollen samples have been processed as standard palynological preparations (see Methods), the potentially damaging oxidation step has not removed or obscured the UAC signal^21^ (Supplementary Fig. S3).

The samples in the younger grassland interval contain a lower proportion of grass pollen compared to the older grassland interval (Fig. 2c), with a higher proportion of tree taxa and representing a more complex vegetation structure^28^. In mixed, tree-covered savannahs shading levels will vary across the landscape, leading to a more heterogeneous UV environment than open grasslands (represented by high Poaceae proportions) or closed forests (low Poaceae proportions), and an accordingly higher within-sample variability of UAC concentrations. Consistent with this expectation, there is a humped relationship between the within-sample UAC standard deviation and the proportion of grass in the pollen sum (Fig. 3c) (see Methods). Therefore, both the mean signal and the within-sample variance are interpretable in the context of a biochemical response to UV inputs over a variety of scales. Further research is required to better understand the impact of confounding factors such as shading on the wider applicability of this proxy.

Despite the sensitivity of the proxy to localised shading and the additional variability that this adds to the UAC signal, useful and interpretable information is recoverable. The results from Lake Bosumtwi show that the expected TSI signal can be tracked via the analysis of small sample sizes (<30 pollen grains per sample) over orbital timescales (10^5^ years). Our successful reconstruction of TSI from standard palynological preparations confirms that sporopollenin chemistry is robust to routine processing procedures (Supplementary Fig. S3)^21^. Therefore the UAC-based approach outlined here can be used to reconstruct UV and TSI from the extensive sporomorph archives that are present in the sediment and rock records, and available ready-processed in palynological laboratories around the world.

The approach reported here opens up a whole new suite of proxies based on fossil sporomorphs. Traditionally the analysis of sporomorphs has been taxon based, effectively using the fossil record as a passive archive, monitoring species occurrence, abundance and diversity over time. However, tracking changes in sporopollenin biogeochemistry indicates that sporomorphs have the potential to be used in a more dynamic way, directly recording past solar inputs. The development and application of these tools could potentially resolve some of the key challenges facing Earth system scientists, such as: quantifying the role of solar irradiance in climate change^1^,^4^, identifying episodes of past ozone collapse^9^, determining the timing and rate of mountain uplift^13^, and calibrating age-depth models against orbital TSI fluctuations. Critically, this proxy can be applied independently of palaeoclimatic and palaeoecological records, allowing for the first time the decoupling of UV (or solar) irradiance, climate, and biotic change.

## Methods

### Modern *Lycopodium* dataset

Samples of *Lycopodium* species (*Lycopodium annotinum, L. magellancium, L. cernua*) were collected from a range of fresh and herbarium sources. These samples span a wide latitudinal transect (54°S to 68°N) and a correspondingly large flux in calculated TSI (Supplementary Table S1).

We used Fourier Transform Infrared (FTIR) microspectroscopy to quantify UAC concentrations, because this provided a non-destructive approach that could be used on small samples^20^, and had been demonstrated to accurately reconstruct trends in sporopollenin UAC concentrations in a range of settings^13^,^14^,^15^,^17^,^18^,^21^,^22^. FTIR microspectroscopy was carried out at The Open University using a Thermo Nicolet (Waltham, MA, USA) FTIR bench unit fitted with a KBr beamsplitter. A Continuum IR-enabled microscope fitted with a 15x reflachromat objective lens and nitrogen-cooled MCT-A detector was interfaced with the bench unit to provide microscopic analysis capability. Atmospheric H_2_O and CO_2_ interference within spectra was countered by purging the entire system (bench unit, microscope and sample stage) with air that has been dried and scrubbed of CO_2_ using a Peak Scientific (Billerica, MA, USA) ML85 purge unit. Analysis was conducted using a microscope aperture of 100 × 100 μm recording the mean of 512 scans per sample with a resolution of 1.928 cm^−1^ wavenumbers. Background spectra were collected immediately after every sample spectrum. Each sample analysis was replicated five times per sample.

UV absorbing compound (UAC) concentrations were quantified by measuring the height of the 1510 cm^−1^ aromatic (C=C) peak, because past research has shown that this records changes in the abundance of UACs in sporopollenin^13^,^15^,^17^,^18^,^20^. The absolute height of peaks in IR spectra relate to the thickness of material being scanned. The 1510 cm^−1^ peak height was therefore normalized against the broad hydroxyl (OH) peak centred on 3300 cm^−1 ^20. Peak heights were measured relative to a linear baseline using ThermoFisher TQ analyst software (Supplementary Table S2).

Estimates of modern TSI were generated using the Apple Macintosh software AnalySeries v2.0^32^ using the Lasker^33^ numerical solution. The timing of *Lycopodium* spore release is not well constrained, and may occur throughout much of the year depending on local climatic and growing conditions^34^. We therefore used the mean annual modelled TSI, taken as the mean across all days of the year at a given latitude, as an expectation to test the *Lycopodium* UAC data against. The dataset of Willis *et al*.^19^, which records concentrations of the UAC *p*-coumaric acid in *Pinus* pollen at a range of latitudes, was used to compare with the latitudinal trend in *Lycopodium* spore UACs. For both datasets UAC levels were regressed onto TSI using linear models.

### Lake Bosumtwi Poaceae dataset

Lake Bosumtwi in southern Ghana (06°32′N, 01°25′W) fills a 1.07 +/− 0.05 Ma meteorite impact crater^27^. The lake is ~10 km in diameter and ~75 m deep, and contains a 294 m thick sedimentary record that has accumulated since the formation of the crater. Multiple cores were taken from Lake Bosumtwi in 2004^29^. The 5B core, from the deepest part of the lake, captured the longest and most expanded sedimentary record taken from Lake Bosumtwi, and has been the focus of previous geochronological^29^ and palynological^28^ research. Samples from this core therefore form the basis of the current study. The upper ~170 kyr of the Bosumtwi sedimentary sequence has been independently dated using a combination of radiocarbon, optically stimulated luminescence, U-series dating and paleomagnetic excursions^29^ (Supplementary Table S3).

Intervals of the Bosumtwi record that are dominated by Poaceae pollen and represent grassland environments were targeted for this study. These samples represent a simple vegetation structure where orbitally-paced variations in UV are expected to be recovered most clearly, but also encompass sufficient variation in Poaceae relative abundance (16 to 96% of the sporomorph sum) to allow the impact of vegetation heterogeneity and localised shading to be investigated. Both the intervals spanning 0–15 kyr (~the Holocene) and ~56–78 kyr are dominated by moist broadleaf forest taxa (e.g., *Alchornea, Celtis, Hymenocardia, Holoptelea, Macaranga*, Mimosoideae, Moraceae, Papilionoideae, *Trema,* and *Uapaca*^28^) and mostly lack Poaceae pollen, and so were excluded from the analysis. Sampling was also limited to the well-dated upper 140 kyr of the core (Supplementary Fig. S2).

The majority of samples in this study are from the dataset documented in Miller and Gosling^28^, which was used to maintain stratigraphic consistency with this previous palynological research. Additional samples were included to fill in gaps and increase sampling resolution as necessary, resulting in 69 samples in total. All samples were prepared using standard palynological processing techniques^35^, including acetolysis (oxidation) since this has been shown to not impact upon sporopollenin (pollen wall) chemistry at normal processing durations^21^. However to confirm this with the current sample set eight samples were processed with and without acetolysis and their peak height ratios compared (Supplementary Fig. S3).

Individual pollen grains were picked from the sediment samples using Narishige MMN-1 and MMO-202ND course and fine control micromanipulators, respectively, and an IM-11–2 pneumatic microinjector, mounted on a Microtec IM-2 inverted microscope. The pollen grains were mounted on ZnSe windows for FTIR analysis, arranged in groups of 8 to 10 grains with 3 replicate groups per sample. FTIR spectra were generated using the same micro-FTIR setup as the *Lycopodium* dataset, with a microscope aperture of 100 × 100 μm recording the mean of 256 scans per sample with a resolution of 1.928 cm^−1^ wavenumbers. As with the *Lycopodium* data, ThermoFisher TQ Analyst software was used to extract the heights of the 1510 cm^−1^ C=C peak and the 3300 cm^−1^ OH peak for each spectrum, relative to a linear baseline. The UAC content was then measured as the C=C/OH (aromatic/hydroxyl) ratio (Supplementary Table S4).

**Figure 2:**
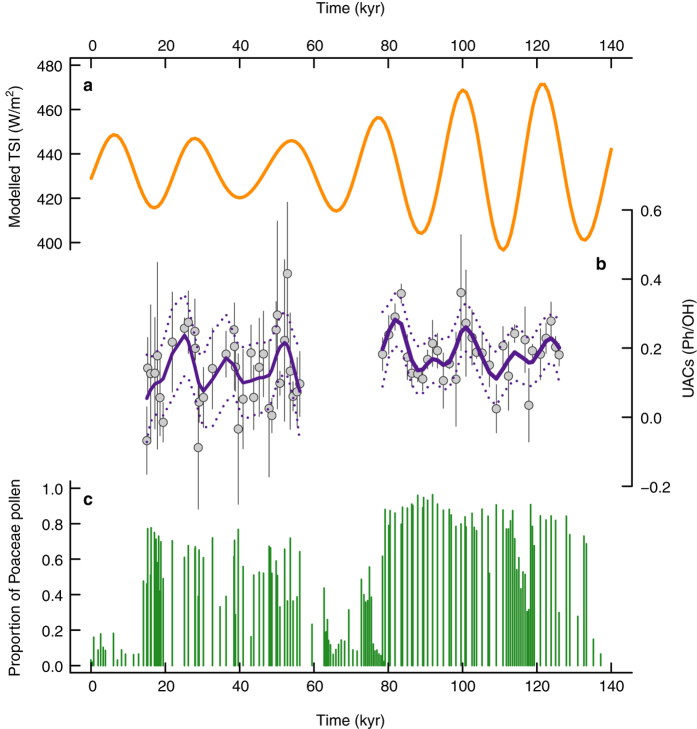
Solar irradiance and pollen chemistry. (**a**) Modelled September total solar irradiance at 6°N^32^. (**b**) UAC concentrations in Poaceae pollen from Lake Bosumtwi. Grey circles are sample means, error bars are one standard deviation. Purple lines are GAM smoothers, based on the sample means. Solid lines show the model fit, dashed lines are 95% confidence intervals. (**c**) Proportion of Poaceae pollen in each sample.

Data analysis was carried out in R v3.2.1^36^, with the packages mgcv v1.8–12^37^ and nlme v3.1–128^38^. Sample age assignments were obtained using the Bacon age-depth model from Shanahan *et al*.^29^, via Bacon v2.2^39^ (Supplementary Fig. S2). In Fig. 2 UAC and Poaceae proportion data are plotted against the Bacon weighted mean age; Supplementary Fig. S2 shows the 95% confidence intervals on the Bacon age assignments.

The growing and flowering phenology of grasses in the west African savannahs is controlled by annual precipitation patterns^30^ (Supplementary Fig. S1), and this in turn will determine the TSI signal the UAC data record. The main dry season occurs in December and January, and the rainy season extends from February to November, interrupted by a shorter dry season in August^30^. Grasses typically start to grow in the first part of the rainy season, and then flower between September and November^30^. Modelled September TSI (=maximum flowering season TSI) was generated using AnalySeries v2.0^32^ using the Lasker^33^ numerical solution.

Both linear and additive models^40^ were fit to the UAC data (described below). In time series such as those analyzed here the assumption of independence among residuals can be violated by temporal autocorrelation^40^. Each model was therefore fit both with and without a continuous time first-order autoregressive process (CAR(1)) correlation structure. The CAR(1) correlation structure was used because this allows for varying temporal distances among residuals, and is therefore appropriate in setting such as this where samples are irregularly spaced in time^41^. The best model was selected using Akaike’s ‘An Information Criterion’ (AIC)^40^,^42^,^43^. AIC incorporates both model fit and model complexity, with more complex models being penalised relative to simpler ones; better models have lower AIC values relative to others in the same candidate set^40^,^42^.

Generalized Additive Model (GAM)^40^,^41^ smoothers were fitted to the mean UAC values to help assess the underlying temporal pattern in the data (Fig. 2b). Rather than fit one smoother across the whole time series, including the ~20 kyr gap in the UAC data, different models were fitted to the two savannah zones. The best model (lowest AIC) for both savannah zones was the model without the CAR(1) correlation structure (AIC of −50.74 versus −54.78 for the younger savannah zone, and −65.09 versus −75.51 for the older savannah zone, for the CAR(1) model and the model with no correlation structure, respectively).

**Figure 3:**
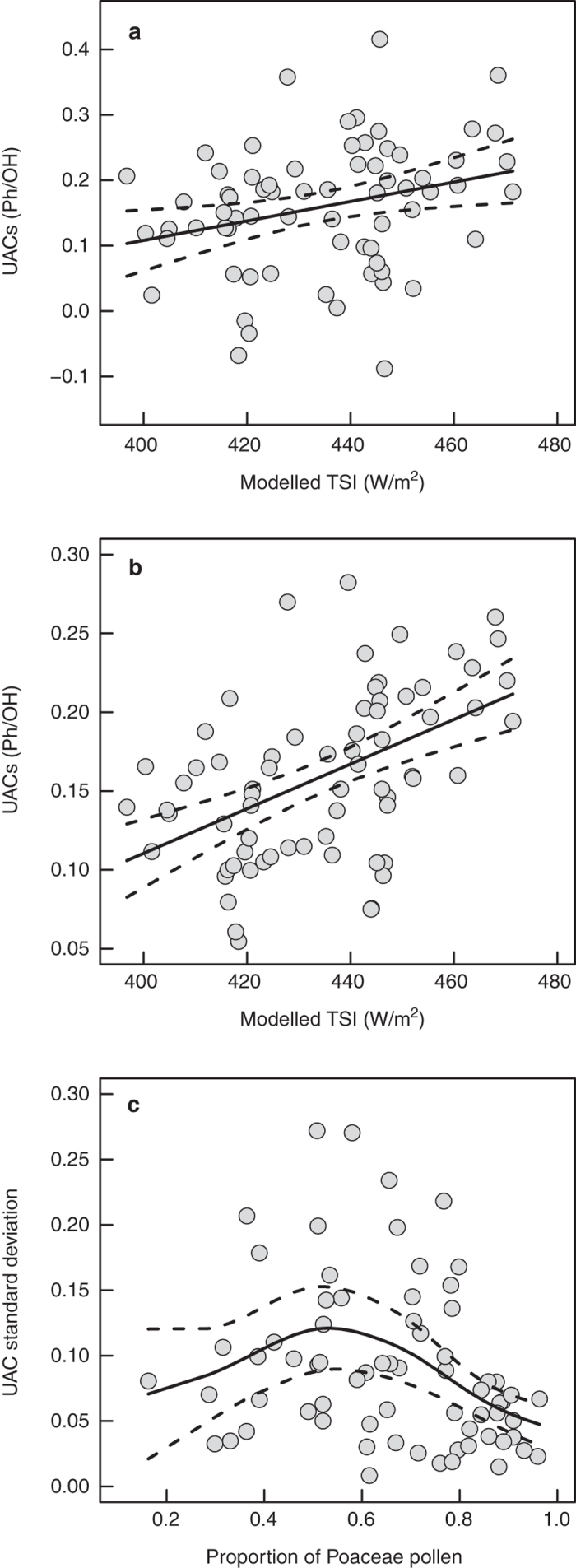
Statistical analysis of Bosumtwi Poaceae UAC data. Solid lines are fitted models, dashed lines are 95% confidence intervals. (**a**) Poaceae UAC concentrations (sample means) plotted against modelled September TSI^32^. Fitted line is a linear multiple regression model, y = −0.57 + 0.00148*TSI + 0.13*Poaceae proportion, *n* = 69, *r*^*2*^ = 0.11, *p* = 0.008. (**b**) GAM-predicted Poaceae UAC concentrations plotted against modelled September TSI^32^. Fitted line is a multiple linear regression model, y = −0.52 + 0.00142*TSI + 0.094*Poaceae proportion, *n* = 69, *r*^*2*^ = 0.34, *p* < 0.0001. (**c**) Within-sample UAC standard deviation plotted against the proportion of Poaceae pollen in each sample. Fitted line is a GAM smoother, *n* = 69, *p* = 0.01, *r*^*2*^ = 0.13.

The relationship between within-sample UAC means and modelled September TSI^32^ was assessed with linear regression. While increased TSI is expected to increase UAC concentrations through enhanced exposure to UV-B, shading from higher stature vegetation will act to decrease UV-B exposure and therefore UAC production^17^. Poaceae proportions (Fig. 2c) provide a proxy for habitat openness, and therefore should correlate positively with UAC concentrations, along with TSI. Three candidate models were therefore considered: one with both TSI and Poaceae proportion as explanatory variables (treated additively, because no interaction between these variables is expected), one with just TSI, and one with just Poaceae proportions. The full model with both explanatory variables was fitted with Generalized Least Squares (GLS)^40^ both with and without the CAR(1) correlation structure. The lowest AIC was for the model without the correlation structure (AIC −100.90 versus −102.90 for the CAR(1) model and the model with no correlation structure, respectively). The three candidate models were therefore fit as Ordinary Least Squares (OLS) linear models with no correlation structure. All explanatory variables were statistically significant in the three models, however the best model was the full model with both TSI and Poaceae proportion (AIC −126.97, Fig. 3a), followed by the TSI only model (AIC −124.07) and then the Poaceae proportion only model (AIC −122.91). The predicted UAC values from the GAM smoother were also used as the response variable with TSI and Poaceae proportion as explanatory variables (Fig. 3b); again all model terms were statistically significant. Figure 3a and b show mean UAC values and the fitted models plotted against TSI, with Poaceae proportion held constant at its mean value (0.66).

Vegetation heterogeneity should also have an effect on recorded UAC levels via heterogeneity of localised shading. In either open or closed (and therefore shaded) environments, which are represented by high or low levels of Poaceae pollen, respectively, all replicate measurements within a sample should be similar. However intermediate levels of Poaceae pollen indicate a more complex savannah environment with varying levels of localised shading, increasing UAC within-sample variance. We therefore modelled the standard deviation of the three replicate UAC measurements for each sample as a function of Poaceae proportion with a GAM smoother with gamma distributed errors (Fig. 3c).

**Figure 4:**
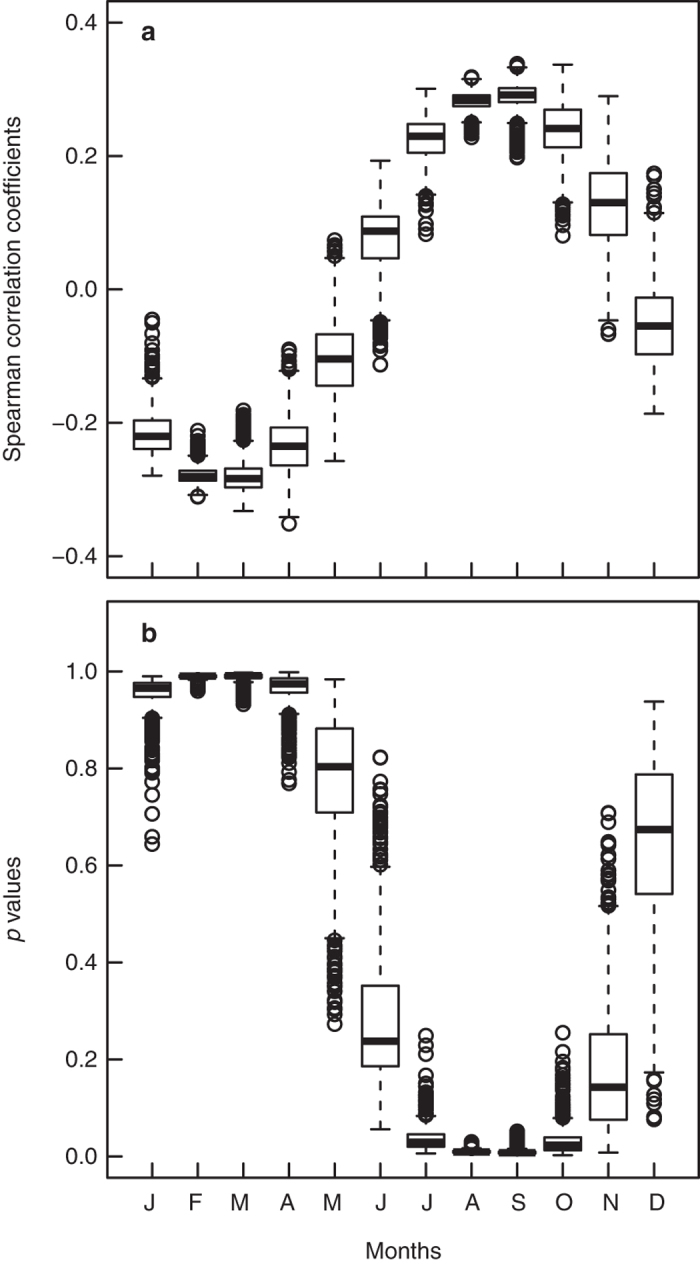
Assessing the impact of age model uncertainty in the pollen chemistry-insolation relationship. Boxplots show the correlation coefficients (**a**) and *p* values (**b**) for correlations of mean within-sample phenolic concentrations against modelled total solar irradiance^32^ for the 21^st^ of each month, from 1000 replicate Markov Chain Monte Carlo (MCMC)-derived age-depth models. The highest correlations occur in August to October, just before and during the Ghanaian grass flowering season^30^.

To test for the effect of age-depth model uncertainty on the UAC-TSI regression (Fig. 3a), 1000 replicate sediment accumulation histories were simulated from the Bacon MCMC sedimentation time estimates, and from these age estimates for each sample obtained. Modelled TSI estimates for the 21^st^ of each month for all 1000 sets of sample ages were then obtained, and a correlation test performed against the UAC sample means. The resultant correlation coefficients and *p* values were plotted up as box plots, with month as the grouping variable (Fig. 4).

## Additional Information

**How to cite this article**: Jardine, P. E. *et al*. Pollen and spores as biological recorders of past ultraviolet irradiance. *Sci. Rep.* **6**, 39269; doi: 10.1038/srep39269 (2016).

**Publisher’s note:** Springer Nature remains neutral with regard to jurisdictional claims in published maps and institutional affiliations.

## Supplementary Material

Supplementary Information

## Figures and Tables

**Figure 1:**
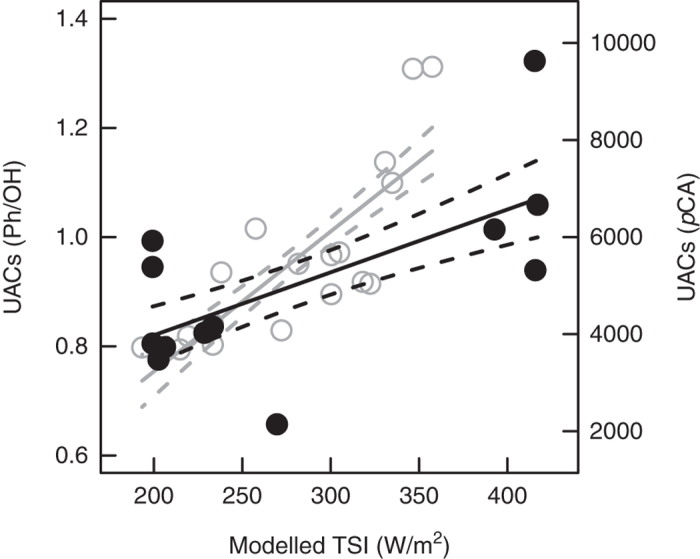
Relationship between modern latitudinal TSI flux and sporomorph chemistry. UAC data for *Lycopodium* spores (black circles and lines) and *Pinus* pollen^19^ (grey open circles and lines), plotted against modelled TSI^32^. Linear regression through both datasets show a significant positive relationship, for *Lycopodium* species y = 0.590 + 0.00115*TSI, *n* = 12, *r*^2^ = 0.36, *p* = 0.023, for *Pinus* y = −2537.51 + 28.84*TSI, *n* = 18, *r*^2^ = 0.62, *p* < 0.0001. Solid lines are fitted models, dashed lines are 95% confidence intervals. Ph/OH = phenolic/hydroxyl ratio from FTIR spectra of *Lycopodium* spores, p-CA = relative abundance of *para*-coumaric acid in *Pinus* pollen^19^.
