# Supplementary material for: Pollen and spores as biological recorders of past ultraviolet irradiance
Source: Sci Rep. 2016 Dec 15;6:39269. doi: 10.1038/srep39269 (PMC5157028; doi:10.1038/srep39269)
Supplement: Supplementary Information [file srep39269-s1.pdf]

Supplementary Information for:

Pollen and spores as biological recorders of past ultraviolet irradiance

Phillip E. Jardine, Wesley T. Fraser, Barry H. Lomax, Mark A. Sephton, Timothy M. Shanahan, Charlotte S. Miller, and William D. Gosling

Supplementary References

44. Ryan, W.B.F. *et al.* Global Multi-Resolution Topography synthesis, *Geochem. Geophys. Geosyst.* **10**, Q03014, doi: 10.1029/2008GC002332 (2009).

Supplementary Tables

Supplementary Table S1. Sample information for modern *Lycopodium* data.

Location	Latitude	Longitude	Species	Sampling	TSI (W/m^2)	*n*	UAC mean	UAC s.d.
Norway	68	18	*L. annotinum*	Field	199.22	3	0.993	0.009
West.Sweden	68	18	*L. annotinum*	Field	199.22	7	0.945	0.051
ANS.Sweden	68	19	*L. annotinum*	Field	199.22	64	0.805	0.095
Greenland.1	67	48	*L. annotinum*	Herbarium	202.56	5	0.776	0.101
Greenland.2	66	52	*L. annotinum*	Herbarium	206.31	7	0.799	0.057
Greenland.3	61	48	*L. annotinum*	Herbarium	228.55	9	0.825	0.096
Greenland.4	60	45	*L. annotinum*	Herbarium	233.32	6	0.836	0.102
India	20	81	*L. annotinum*	Herbarium	392.67	6	1.014	0.086
Malaysia	4	102	*L. annotinum*	Herbarium	415.81	7	0.939	0.136
Ecuador	-1	-78	*L. magellancium*	Herbarium	417.07	22	1.059	0.162
SE.Asia	-6	100	*L. cernua*	Field	415.43	5	1.323	0.199
South.Georgia	-54	-37	*L. magellancium*	Field	269.62	6	0.657	0.079

TSI is the modelled mean annual total solar irradiance[32], *n* is the number of samples from each locality, UAC mean is the mean aromatic/OH ratio, and UAC s.d. is the standard deviation. See Supplementary Table S2 for the sample UAC values.

Supplementary Table S2. UAC data for modern *Lycopodium* data.

Sample	Location	UACs
Norway.1	Norway	1.0036
Norway.2	Norway	0.9854
Norway.3	Norway	0.9906
West.Sweden.1	West.Sweden	1.0246
West.Sweden.2	West.Sweden	0.8822
West.Sweden.3	West.Sweden	0.9496
West.Sweden.4	West.Sweden	0.9153
West.Sweden.5	West.Sweden	0.9709
West.Sweden.6	West.Sweden	0.9792
West.Sweden.7	West.Sweden	0.8949
ANS.Sweden.1	ANS.Sweden	0.8259
ANS.Sweden.2	ANS.Sweden	1.0599
ANS.Sweden.3	ANS.Sweden	0.7979
ANS.Sweden.4	ANS.Sweden	0.8539
ANS.Sweden.5	ANS.Sweden	0.7012
ANS.Sweden.6	ANS.Sweden	0.7883
ANS.Sweden.7	ANS.Sweden	0.8014
ANS.Sweden.8	ANS.Sweden	0.8549
ANS.Sweden.9	ANS.Sweden	0.8443
ANS.Sweden.10	ANS.Sweden	0.7717
ANS.Sweden.11	ANS.Sweden	0.6952
ANS.Sweden.12	ANS.Sweden	0.69
ANS.Sweden.13	ANS.Sweden	0.7528
ANS.Sweden.14	ANS.Sweden	0.8187
ANS.Sweden.15	ANS.Sweden	0.7959
ANS.Sweden.16	ANS.Sweden	0.8471
ANS.Sweden.17	ANS.Sweden	0.8567
ANS.Sweden.18	ANS.Sweden	0.5905
ANS.Sweden.19	ANS.Sweden	0.6744
ANS.Sweden.20	ANS.Sweden	0.7667
ANS.Sweden.21	ANS.Sweden	0.7647
ANS.Sweden.22	ANS.Sweden	0.8198
ANS.Sweden.23	ANS.Sweden	0.8296
ANS.Sweden.24	ANS.Sweden	0.9329
ANS.Sweden.25	ANS.Sweden	0.9205
ANS.Sweden.26	ANS.Sweden	0.9548
ANS.Sweden.27	ANS.Sweden	0.8706
ANS.Sweden.28	ANS.Sweden	0.8936
ANS.Sweden.29	ANS.Sweden	0.9121
ANS.Sweden.30	ANS.Sweden	0.9086
ANS.Sweden.31	ANS.Sweden	0.9768
ANS.Sweden.32	ANS.Sweden	0.7849
ANS.Sweden.33	ANS.Sweden	0.9618
ANS.Sweden.34	ANS.Sweden	0.9684
ANS.Sweden.35	ANS.Sweden	0.9025
ANS.Sweden.36	ANS.Sweden	0.7746
ANS.Sweden.37	ANS.Sweden	0.7361
ANS.Sweden.38	ANS.Sweden	0.6783
ANS.Sweden.39	ANS.Sweden	0.669
ANS.Sweden.40	ANS.Sweden	0.6929
ANS.Sweden.41	ANS.Sweden	0.8237
ANS.Sweden.42	ANS.Sweden	0.6857
ANS.Sweden.43	ANS.Sweden	0.6077
ANS.Sweden.44	ANS.Sweden	0.7761
ANS.Sweden.45	ANS.Sweden	0.7998
ANS.Sweden.46	ANS.Sweden	0.7286
ANS.Sweden.47	ANS.Sweden	0.8333
ANS.Sweden.48	ANS.Sweden	0.7419
ANS.Sweden.49	ANS.Sweden	0.8497
ANS.Sweden.50	ANS.Sweden	0.6635

ANS.Sweden.51	ANS.Sweden	0.7639
ANS.Sweden.52	ANS.Sweden	0.7208
ANS.Sweden.53	ANS.Sweden	0.6695
ANS.Sweden.54	ANS.Sweden	0.7689
ANS.Sweden.55	ANS.Sweden	0.8414
ANS.Sweden.56	ANS.Sweden	0.8206
ANS.Sweden.57	ANS.Sweden	0.9701
ANS.Sweden.58	ANS.Sweden	0.8988
ANS.Sweden.59	ANS.Sweden	0.8232
ANS.Sweden.60	ANS.Sweden	0.7506
ANS.Sweden.61	ANS.Sweden	0.8277
ANS.Sweden.62	ANS.Sweden	0.8331
ANS.Sweden.63	ANS.Sweden	0.7704
ANS.Sweden.64	ANS.Sweden	0.8111
Greenland.1.1	Greenland.1	0.75
Greenland.1.2	Greenland.1	0.6274
Greenland.1.3	Greenland.1	0.9057
Greenland.1.4	Greenland.1	0.7963
Greenland.1.5	Greenland.1	0.7988
Greenland.2.1	Greenland.2	0.7899
Greenland.2.2	Greenland.2	0.8472
Greenland.2.3	Greenland.2	0.7879
Greenland.2.4	Greenland.2	0.8262
Greenland.2.5	Greenland.2	0.6863
Greenland.2.6	Greenland.2	0.7962
Greenland.2.7	Greenland.2	0.8562
Greenland.3.1	Greenland.3	0.9975
Greenland.3.2	Greenland.3	0.7636
Greenland.3.3	Greenland.3	0.8274
Greenland.3.4	Greenland.3	0.739
Greenland.3.5	Greenland.3	0.7242
Greenland.3.6	Greenland.3	0.7192
Greenland.3.7	Greenland.3	0.8938
Greenland.3.8	Greenland.3	0.8903
Greenland.3.9	Greenland.3	0.867
Greenland.4.1	Greenland.4	0.8866
Greenland.4.2	Greenland.4	0.8844
Greenland.4.3	Greenland.4	0.8907
Greenland.4.4	Greenland.4	0.9126
Greenland.4.5	Greenland.4	0.6455
Greenland.4.6	Greenland.4	0.7952
India.1	India	1.025
India.2	India	1.0429
India.3	India	1.0131
India.4	India	0.8661
India.5	India	1.0055
India.6	India	1.1322
Malaysia.1	Malaysia	0.8689
Malaysia.2	Malaysia	1.1747
Malaysia.3	Malaysia	0.8429
Malaysia.4	Malaysia	1.0813
Malaysia.5	Malaysia	0.9375
Malaysia.6	Malaysia	0.8256
Malaysia.7	Malaysia	0.8449
Ecuador.1	Ecuador	1.0107
Ecuador.2	Ecuador	1.1037
Ecuador.3	Ecuador	1.0511
Ecuador.4	Ecuador	0.836
Ecuador.5	Ecuador	0.9486
Ecuador.6	Ecuador	1.3016
Ecuador.7	Ecuador	1.2821
Ecuador.8	Ecuador	1.0791
Ecuador.9	Ecuador	0.9376
Ecuador.10	Ecuador	0.869

Ecuador.11	Ecuador	0.9961
Ecuador.12	Ecuador	1.221
Ecuador.13	Ecuador	1.1416
Ecuador.14	Ecuador	0.9466
Ecuador.15	Ecuador	1.1378
Ecuador.16	Ecuador	1.0826
Ecuador.17	Ecuador	0.8007
Ecuador.18	Ecuador	0.8162
Ecuador.19	Ecuador	0.9417
Ecuador.20	Ecuador	1.255
Ecuador.21	Ecuador	1.2883
Ecuador.22	Ecuador	1.2556
SE.Asia.1	SE.Asia	1.2015
SE.Asia.2	SE.Asia	1.31
SE.Asia.3	SE.Asia	1.6421
SE.Asia.4	SE.Asia	1.3404
SE.Asia.5	SE.Asia	1.1193
South.Georgia.1	South.Georgia	0.5405
South.Georgia.2	South.Georgia	0.7607
South.Georgia.3	South.Georgia	0.6721
South.Georgia.4	South.Georgia	0.6436
South.Georgia.5	South.Georgia	0.7188
South.Georgia.6	South.Georgia	0.6071

UACs are the aromatic/OH values for each sample, and are the mean of 5 replicate micro-FTIR measurements. Location UAC means and standard deviations are given in Supplementary Table S1.

Supplementary Table S3. Sample information for fossil Poaceae data from Lake Bosumtwi.

Sample	Depth (mblf)	Weighted mean age	Lower 95 age	Upper 95 age	Poaceae prop.	TSI (W/m^2)	UAC mean	UAC s.d.
2H_2_60	5.1	15002.26	14834.06	15350.43	0.46	418.36	-0.068	0.098
2H_2_70	5.2	15231.78	15071.56	15539.04	0.77	417.80	0.142	0.089
2H_2_105	5.55	16034.96	15871.04	16210.78	0.51	416.36	0.126	0.199
3H_1_10	6.1	17178.32	16964.93	17385.04	0.53	415.78	0.127	0.162
3H_1_70	6.7	17849.32	17694.94	17988.37	0.58	416.28	0.178	0.270
3H_2_10	7.6	18545.38	18382.17	18708.77	0.42	417.41	0.057	0.110
3H_2_70	8.2	19399.64	19166.71	19627.18	0.49	419.59	-0.015	0.057
4H_1_0	9	21854.79	21634.06	22057.13	0.70	429.27	0.217	0.145
4H_1_100	10	25135.76	24746.21	25399.45	0.61	442.83	0.257	0.030
4H_2_51	11	26169.43	25979.13	26345.89	0.68	445.44	0.275	0.091
5H_1_0	12	27949.07	27841.49	28043.42	0.66	447.16	0.249	0.094
5H_1_100	13	28052.59	27946.16	28188.89	0.67	447.14	0.199	0.033
5H_2_49	13.99	28843.86	28580.35	29061.83	0.39	446.58	-0.088	0.179
6H_1_0	15	29064.82	28777.65	29476.25	0.65	446.30	0.044	0.059
6H_1_100	16	30236.13	29671.49	30996.29	0.61	444.07	0.057	0.087
6H_1_130	16.3	32672.88	32200.45	33118.10	0.72	436.48	0.141	0.117
6H_2_25	16.75	36330.12	35478.38	36919.44	0.39	424.83	0.182	0.066
6H_2_85	17.35	38470.96	37318.56	39341.81	0.62	421.08	0.253	0.008
6H_2_125	17.75	38596.80	37440.59	39610.39	0.71	420.96	0.205	0.126
7H_1_10	18.1	38861.10	37826.12	39968.24	0.29	420.75	0.145	0.070
7H_1_50	18.5	39614.53	38734.43	41040.53	0.77	420.38	-0.034	0.218
7H_1_110	19.1	40913.18	39532.15	43107.26	0.56	420.61	0.052	0.144
7H_2_20	19.7	43060.79	40533.10	45408.81	0.16	423.21	0.186	0.081
7H_2_40	19.9	43772.87	40745.23	46586.14	0.51	424.56	0.057	0.093
7H_2_100	20.5	45258.43	42795.73	47748.06	0.53	428.01	0.144	0.142
8H_1_0	21	46392.07	43803.72	48803.98	0.52	431.00	0.183	0.124
8H_1_80	21.8	47911.70	46050.17	50257.51	0.67	435.28	0.025	0.198
8H_1_120	22.2	48675.27	46593.53	51328.03	0.52	437.38	0.005	0.050
8H_2_30	22.8	49813.30	47160.12	53195.59	0.59	440.34	0.253	0.082
8H_2_49	22.99	50161.72	47265.42	53985.84	0.51	441.18	0.296	0.272
8H_2_84.5	23.345	50833.87	47935.08	54557.52	0.33	442.68	0.098	0.035
9H_1_0	24	52090.09	48541.51	56249.86	0.66	444.82	0.222	0.234
9H_1_40	24.4	52847.09	49450.30	56828.26	0.36	445.66	0.415	0.207
9H_1_80	24.8	53612.69	49971.47	57636.68	0.72	446.11	0.133	0.168
9H_1_120	25.2	54360.52	50660.60	58390.89	0.36	446.07	0.060	0.042
9H_2_30	25.8	55457.98	51577.73	59428.86	0.39	445.12	0.074	0.099
9H_2_70	26.2	56179.04	52332.06	60198.60	0.64	443.95	0.096	0.094
12H_2_55	35.05	78454.79	73205.68	83768.98	0.62	455.44	0.182	0.048
13H_1_5	36.05	80136.98	74765.08	85463.98	0.79	449.54	0.239	0.056
13H_1_105	37.05	81824.64	76513.66	87222.16	0.86	439.55	0.290	0.038
13H_2_54	38.05	83489.25	78199.97	88777.68	0.80	427.82	0.358	0.028
14H_1_5	39.05	85127.41	79861.96	90453.75	0.89	416.65	0.174	0.065
14H_1_85	39.85	86323.34	81240.10	91576.02	0.91	410.17	0.127	0.038
14H_2_54	41.05	87922.12	82587.49	93312.02	0.96	404.92	0.125	0.023
15H_1_5	42.05	89274.03	83840.71	94710.11	0.89	404.52	0.111	0.034
15H_1_105	43.05	90588.90	85044.22	96051.42	0.93	407.75	0.167	0.028
15H_2_55	44.05	91920.07	86361.43	97447.52	0.96	414.65	0.214	0.067
16H_1_5	45.05	93241.66	87635.02	98719.79	0.91	424.29	0.192	0.050
16H_1_125	46.25	94825.98	89359.61	100200.21	0.88	438.11	0.106	0.080
16H_2_96	47.45	96420.00	90987.70	101714.97	0.88	451.96	0.155	0.015
17H_1_85	48.84	98232.22	92924.90	103399.49	0.78	464.17	0.110	0.136
17H_2_36	49.85	99579.55	94354.41	104559.45	0.80	468.54	0.361	0.168
17H_2_136	50.85	100924.00	95856.16	105852.25	0.78	468.00	0.272	0.154
18H_1_105	52.05	102545.36	97626.49	107435.56	0.86	460.42	0.231	0.080
18H_2_35	52.85	103751.94	98777.71	108730.30	0.76	450.77	0.188	0.018
18H_2_125	53.75	105260.80	100158.02	110375.23	0.88	435.60	0.186	0.064
19H_1_85	54.85	107227.54	101808.66	112699.02	0.52	415.51	0.151	0.063
19H_2_35	55.85	109082.80	103542.19	114694.86	0.91	401.57	0.024	0.070
19H_2_115	56.65	110879.07	105190.38	116504.90	0.88	396.75	0.206	0.056
20H_1_25	57.25	112355.11	106553.36	118091.17	0.77	400.37	0.119	0.099

20H_1_85	57.85	114029.53	107836.40	119874.65	0.72	411.96	0.242	0.026
20H_2_36	58.85	116828.42	110801.73	122562.63	0.51	441.47	0.224	0.095
20H_2_76	59.25	117841.81	112037.19	123539.80	0.32	452.13	0.034	0.106
20H_2_116	59.65	118804.48	113218.67	124230.91	0.78	460.75	0.192	0.019
21H_1_65	60.65	120953.51	115875.35	126017.19	0.84	471.39	0.182	0.074
21H_1_145	61.45	122472.25	117441.91	127466.59	0.82	470.28	0.228	0.044
21H_2_76	62.25	123829.42	118460.77	129283.74	0.84	463.55	0.278	0.055
22H_1_0	63	125053.58	119274.54	131034.85	0.82	453.94	0.203	0.031
22H_1_60	63.6	125988.05	120241.26	132018.07	0.30	445.17	0.181	0.033

Weighted mean age, lower 95 age and upper 95 age are the reconstructed weighted mean and 95% credible interval ages from the BACON age depth model[39]. Poaceae proportion is the proportion of Poaceae in the pollen sum[29], TSI is the modelled total solar irradiance for 21st September[32], UAC mean is the mean aromatic/OH ratio across three replicates, and UAC s.d. is the standard deviation. See Supplementary Table S4 for the UAC values from each replicate.

Supplementary Table S4. UAC data for fossil Poaceae data from Lake Bosumtwi.

Replicate	Sample	UACs
2H_2_60_1	2H_2_60	0.024
2H_2_60_2	2H_2_60	-0.171
2H_2_60_3	2H_2_60	-0.056
2H_2_70_1	2H_2_70	0.064
2H_2_70_2	2H_2_70	0.123
2H_2_70_3	2H_2_70	0.239
2H_2_105_1	2H_2_105	-0.103
2H_2_105_2	2H_2_105	0.257
2H_2_105_3	2H_2_105	0.224
3H_1_10_1	3H_1_10	-0.031
3H_1_10_2	3H_1_10	0.122
3H_1_10_3	3H_1_10	0.292
3H_1_70_1	3H_1_70	-0.130
3H_1_70_2	3H_1_70	0.289
3H_1_70_3	3H_1_70	0.375
3H_2_10_1	3H_2_10	-0.026
3H_2_10_2	3H_2_10	0.182
3H_2_10_3	3H_2_10	0.014
3H_2_70_1	3H_2_70	0.033
3H_2_70_2	3H_2_70	0.000
3H_2_70_3	3H_2_70	-0.078
4H_1_0_1	4H_1_0	0.329
4H_1_0_2	4H_1_0	0.269
4H_1_0_3	4H_1_0	0.054
4H_1_100_1	4H_1_100	0.240
4H_1_100_2	4H_1_100	0.240
4H_1_100_3	4H_1_100	0.292
4H_2_51_1	4H_2_51	0.219
4H_2_51_2	4H_2_51	0.226
4H_2_51_3	4H_2_51	0.379
5H_1_0_1	5H_1_0	0.344
5H_1_0_2	5H_1_0	0.244
5H_1_0_3	5H_1_0	0.157
5H_1_100_1	5H_1_100	0.167
5H_1_100_2	5H_1_100	0.233
5H_1_100_3	5H_1_100	0.198
5H_2_49_1	5H_2_49	0.057
5H_2_49_2	5H_2_49	-0.034
5H_2_49_3	5H_2_49	-0.287
6H_1_0_1	6H_1_0	0.108
6H_1_0_2	6H_1_0	-0.007
6H_1_0_3	6H_1_0	0.030
6H_1_100_1	6H_1_100	-0.040
6H_1_100_2	6H_1_100	0.129
6H_1_100_3	6H_1_100	0.081
6H_1_130_1	6H_1_130	0.053
6H_1_130_2	6H_1_130	0.096
6H_1_130_3	6H_1_130	0.274
6H_2_25_1	6H_2_25	0.106
6H_2_25_2	6H_2_25	0.217
6H_2_25_3	6H_2_25	0.224
6H_2_85_1	6H_2_85	0.257
6H_2_85_2	6H_2_85	0.259
6H_2_85_3	6H_2_85	0.243
6H_2_125_1	6H_2_125	0.150
6H_2_125_2	6H_2_125	0.115
6H_2_125_3	6H_2_125	0.349
7H_1_10_1	7H_1_10	0.177
7H_1_10_2	7H_1_10	0.193
7H_1_10_3	7H_1_10	0.065

7H_1_50_1	7H_1_50	-0.081
7H_1_50_2	7H_1_50	0.203
7H_1_50_3	7H_1_50	-0.225
7H_1_110_1	7H_1_110	-0.099
7H_1_110_2	7H_1_110	0.188
7H_1_110_3	7H_1_110	0.068
7H_2_20_1	7H_2_20	0.098
7H_2_20_2	7H_2_20	0.256
7H_2_20_3	7H_2_20	0.204
7H_2_40_1	7H_2_40	0.102
7H_2_40_2	7H_2_40	0.119
7H_2_40_3	7H_2_40	-0.050
7H_2_100_1	7H_2_100	0.226
7H_2_100_2	7H_2_100	-0.020
7H_2_100_3	7H_2_100	0.227
8H_1_0_1	8H_1_0	0.046
8H_1_0_2	8H_1_0	0.215
8H_1_0_3	8H_1_0	0.288
8H_1_80_1	8H_1_80	-0.075
8H_1_80_2	8H_1_80	0.253
8H_1_80_3	8H_1_80	-0.103
8H_1_120_1	8H_1_120	-0.051
8H_1_120_2	8H_1_120	0.020
8H_1_120_3	8H_1_120	0.046
8H_2_30_1	8H_2_30	0.283
8H_2_30_2	8H_2_30	0.316
8H_2_30_3	8H_2_30	0.161
8H_2_49_1	8H_2_49	0.135
8H_2_49_2	8H_2_49	0.143
8H_2_49_3	8H_2_49	0.610
8H_2_84.5_1	8H_2_84.5	0.073
8H_2_84.5_2	8H_2_84.5	0.138
8H_2_84.5_3	8H_2_84.5	0.084
9H_1_0_1	9H_1_0	0.047
9H_1_0_2	9H_1_0	0.132
9H_1_0_3	9H_1_0	0.488
9H_1_40_1	9H_1_40	0.180
9H_1_40_2	9H_1_40	0.500
9H_1_40_3	9H_1_40	0.567
9H_1_80_1	9H_1_80	-0.038
9H_1_80_2	9H_1_80	0.139
9H_1_80_3	9H_1_80	0.299
9H_1_120_1	9H_1_120	0.063
9H_1_120_2	9H_1_120	0.101
9H_1_120_3	9H_1_120	0.017
9H_2_30_1	9H_2_30	0.034
9H_2_30_2	9H_2_30	0.186
9H_2_30_3	9H_2_30	0.000
9H_2_70_1	9H_2_70	0.200
9H_2_70_2	9H_2_70	0.016
9H_2_70_3	9H_2_70	0.073
12H_2_55_1	12H_2_55	0.185
12H_2_55_2	12H_2_55	0.229
12H_2_55_3	12H_2_55	0.133
13H_1_5_1	13H_1_5	0.275
13H_1_5_2	13H_1_5	0.174
13H_1_5_3	13H_1_5	0.268
13H_1_105_1	13H_1_105	0.329
13H_1_105_2	13H_1_105	0.253
13H_1_105_3	13H_1_105	0.288
13H_2_54_1	13H_2_54	0.390
13H_2_54_2	13H_2_54	0.342
13H_2_54_3	13H_2_54	0.341
14H_1_5_1	14H_1_5	0.127

14H_1_5_2	14H_1_5	0.147
14H_1_5_3	14H_1_5	0.248
14H_1_85_1	14H_1_85	0.143
14H_1_85_2	14H_1_85	0.155
14H_1_85_3	14H_1_85	0.084
14H_2_54_1	14H_2_54	0.104
14H_2_54_2	14H_2_54	0.150
14H_2_54_3	14H_2_54	0.123
15H_1_5_1	15H_1_5	0.145
15H_1_5_2	15H_1_5	0.110
15H_1_5_3	15H_1_5	0.077
15H_1_105_1	15H_1_105	0.163
15H_1_105_2	15H_1_105	0.141
15H_1_105_3	15H_1_105	0.196
15H_2_55_1	15H_2_55	0.137
15H_2_55_2	15H_2_55	0.246
15H_2_55_3	15H_2_55	0.259
16H_1_5_1	16H_1_5	0.182
16H_1_5_2	16H_1_5	0.247
16H_1_5_3	16H_1_5	0.148
16H_1_125_1	16H_1_125	0.045
16H_1_125_2	16H_1_125	0.196
16H_1_125_3	16H_1_125	0.075
16H_2_96_1	16H_2_96	0.153
16H_2_96_2	16H_2_96	0.141
16H_2_96_3	16H_2_96	0.171
17H_1_85_1	17H_1_85	0.067
17H_1_85_2	17H_1_85	0.000
17H_1_85_3	17H_1_85	0.262
17H_2_36_1	17H_2_36	0.167
17H_2_36_2	17H_2_36	0.458
17H_2_36_3	17H_2_36	0.457
17H_2_136_1	17H_2_136	0.300
17H_2_136_2	17H_2_136	0.410
17H_2_136_3	17H_2_136	0.106
18H_1_105_1	18H_1_105	0.314
18H_1_105_2	18H_1_105	0.224
18H_1_105_3	18H_1_105	0.154
18H_2_35_1	18H_2_35	0.176
18H_2_35_2	18H_2_35	0.208
18H_2_35_3	18H_2_35	0.180
18H_2_125_1	18H_2_125	0.157
18H_2_125_2	18H_2_125	0.141
18H_2_125_3	18H_2_125	0.259
19H_1_85_1	19H_1_85	0.216
19H_1_85_2	19H_1_85	0.145
19H_1_85_3	19H_1_85	0.091
19H_2_35_1	19H_2_35	0.046
19H_2_35_2	19H_2_35	0.081
19H_2_35_3	19H_2_35	-0.054
19H_2_115_1	19H_2_115	0.158
19H_2_115_2	19H_2_115	0.268
19H_2_115_3	19H_2_115	0.194
20H_1_25_1	20H_1_25	0.044
20H_1_25_2	20H_1_25	0.081
20H_1_25_3	20H_1_25	0.232
20H_1_85_1	20H_1_85	0.214
20H_1_85_2	20H_1_85	0.247
20H_1_85_3	20H_1_85	0.265
20H_2_36_1	20H_2_36	0.179
20H_2_36_2	20H_2_36	0.161
20H_2_36_3	20H_2_36	0.333
20H_2_76_1	20H_2_76	-0.085
20H_2_76_2	20H_2_76	0.119

20H_2_76_3	20H_2_76	0.070
20H_2_116_1	20H_2_116	0.201
20H_2_116_2	20H_2_116	0.171
20H_2_116_3	20H_2_116	0.205
21H_1_65_1	21H_1_65	0.216
21H_1_65_2	21H_1_65	0.098
21H_1_65_3	21H_1_65	0.233
21H_1_145_1	21H_1_145	0.185
21H_1_145_2	21H_1_145	0.227
21H_1_145_3	21H_1_145	0.273
21H_2_76_1	21H_2_76	0.339
21H_2_76_2	21H_2_76	0.264
21H_2_76_3	21H_2_76	0.232
22H_1_0_1	22H_1_0	0.236
22H_1_0_2	22H_1_0	0.199
22H_1_0_3	22H_1_0	0.174
22H_1_60_1	22H_1_60	0.185
22H_1_60_2	22H_1_60	0.146
22H_1_60_3	22H_1_60	0.211

UACs are the aromatic/OH values for each replicate. Sample UAC means and standard deviations are provided in **S**upplementary Table S3.

Supplementary Figures

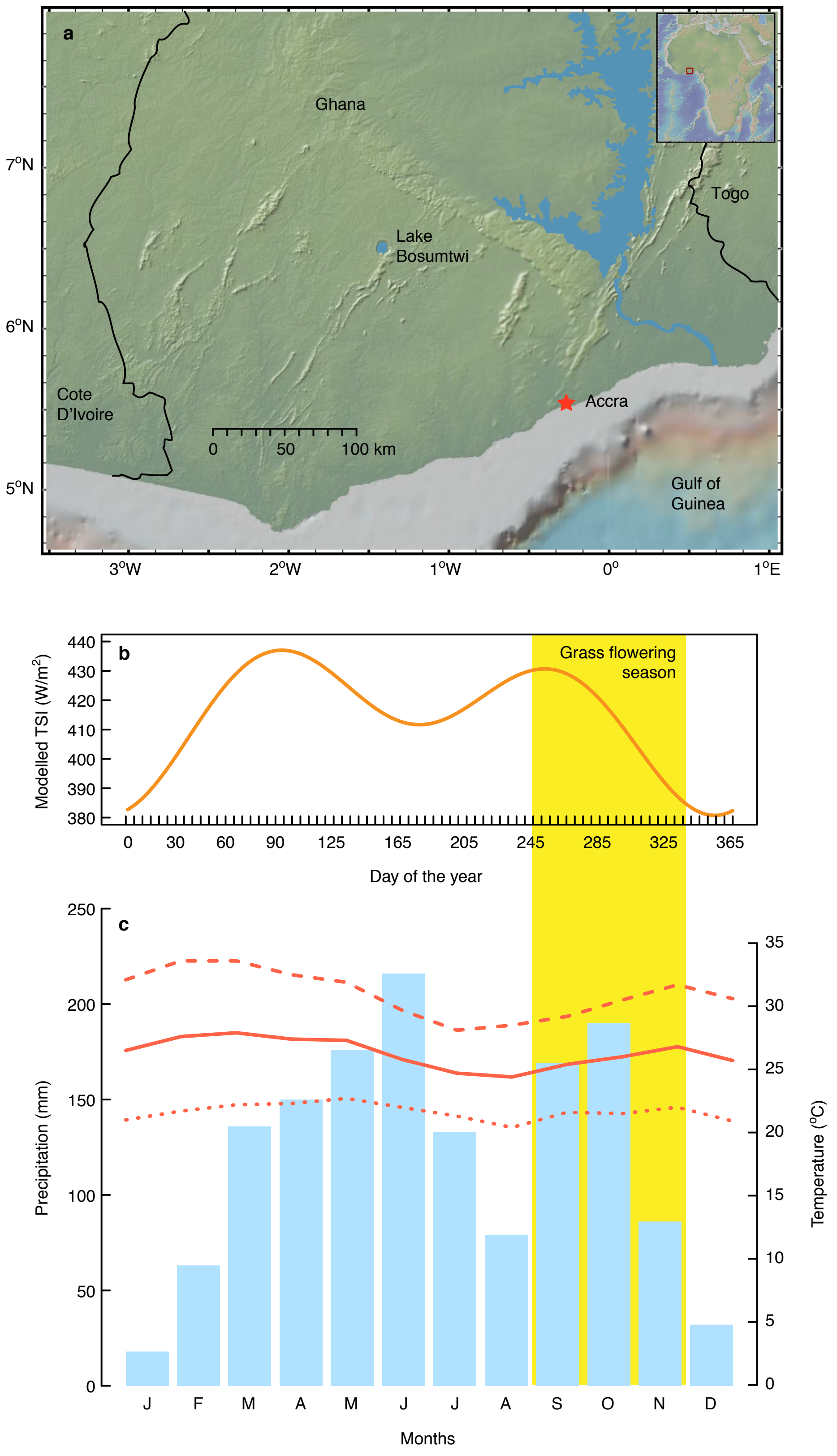

Supplementary Figure S1. Lake Bosumtwi location and modern Ghanaian solar irradiance and climate. **a**, Location map showing the position of Lake Bosumtwi. Basemap created using

GeoMapApp v. 3.6.3, http://www.geomapapp.org, using the Global Multi-Resolution Topography (GMRT) data compilation[44]. **b**, Modelled solar irradiance at 6°N[32]. **c**, Monthly precipitation (blue bars) and minimum, mean and maximum temperature (dotted, solid and dashed red lines, respectively) for the city of Kumasi, ~30 km NW of Lake Bosumtwi (climate data from http://en.climate-data.org/). Yellow box shows Ghanaian grass flowering season[30].

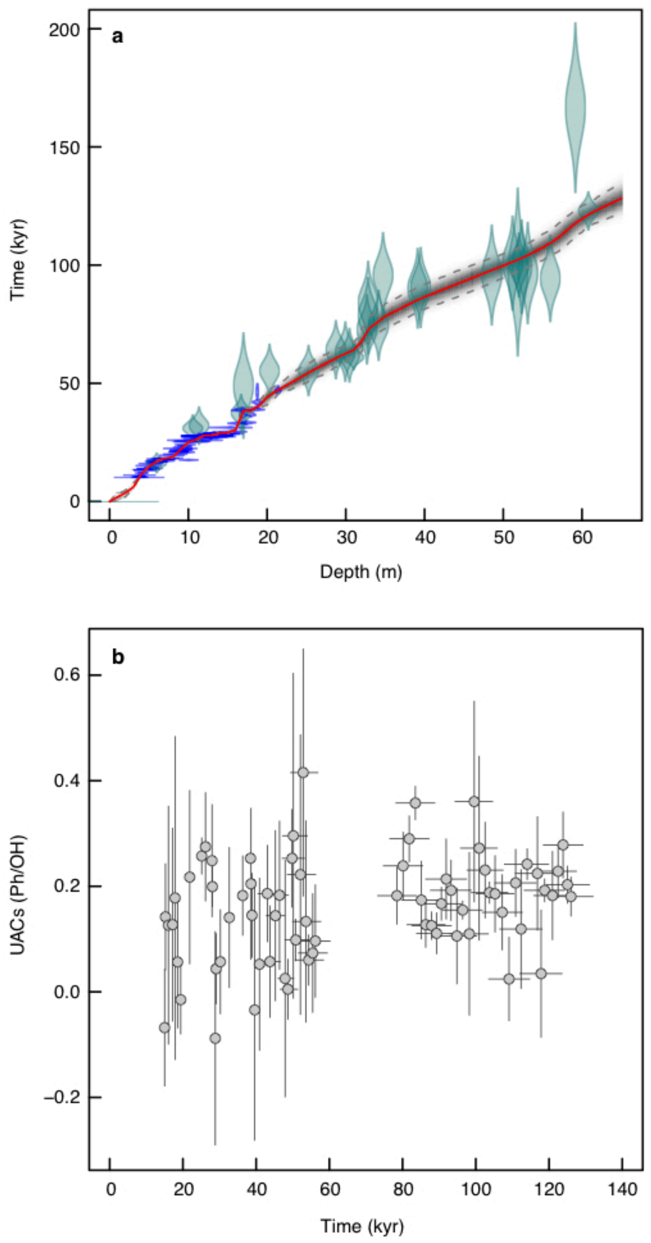

Supplementary Figure S2. Age-depth model for Lake Bosumtwi. a, Bayesian-calibrated age-depth model for the Lake Bosumtwi sediment sequence, calculated using the Bacon software package[39]. Blue symbols are the age estimates with associated uncertainties, solid red line is the median Markov Chain Monte Carlo (MCMC)-derived age-depth model, and the dotted black lines are the 95% confidence intervals. **b**, Age-depth model uncertainty in sample age estimation. UAC concentrations in Poaceae pollen from Lake Bosumtwi. Solid circles are the sample mean phenolic concentrations, plotted against the median sample age from the Bacon age-depth model. Vertical and horizontal lines are 95% error bars for the phenolic concentrations and sample ages, respectively.

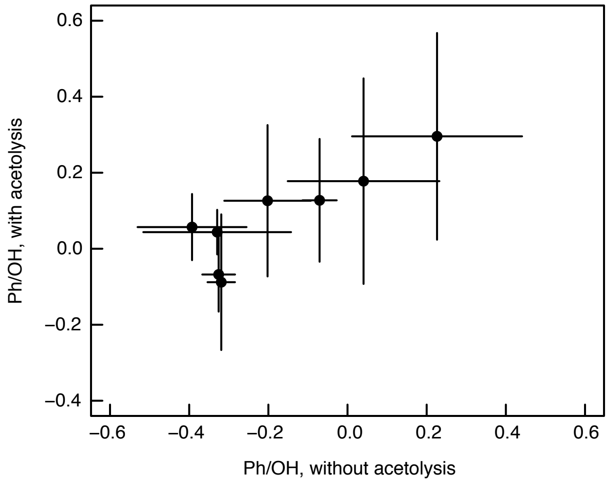

Supplementary Figure S3. Comparison of phenolic concentrations in samples with and without acetolysis (oxidation) as part of the processing procedure. Circles are the mean of three replicates, vertical lines are 1 standard deviation. For correlation among means, $n = 8$, Spearman's $r = 0.87$, $p = 0.005$.
